# Development of a Lung Vacancy Mouse Model through CRISPR/Cas9-Mediated Deletion of Thyroid Transcription Factor 1 Exon 2

**DOI:** 10.3390/cells11233874

**Published:** 2022-12-01

**Authors:** Lihua Zhao, Meishuang Li, Zhibao Yin, Limin Lv, Meng Zhou, Yixi Wang, Manling Zhang, Tianxu Guo, Xiyun Guo, Han Liu, Linxin Cheng, Xiubin Liang, Shuguang Duo, Rongfeng Li

**Affiliations:** 1 Jiangsu Key Laboratory of Xenotransplantation, Nanjing Medical University, Nanjing 211166, China; 2 Key Laboratory of Targeted Intervention of Cardiovascular Disease, Collaborative Innovation Center for Cardiovascular Disease Translational Medicine, Nanjing Medical University, Nanjing 211166, China; 3 Laboratory Animal Center, Institute of Zoology, Chinese Academy of Sciences, Beijing 100101, China; 4 Department of Pathophysiology, Nanjing Medical University, Nanjing 211166, China

**Keywords:** *TTF-1*, CRISPR/Cas9, lung, developmental niche

## Abstract

A developmental niche vacancy in host embryos is necessary for stem cell complementation-based organ regeneration (SCOG). Thyroid transcription factor 1 (TTF-1) is a tissue-specific transcription factor that regulates the embryonic development and differentiation of the thyroid and, more importantly, lungs; thus, it has been considered as a master gene to knockout in order to develop a lung vacancy host. *TTF-1* knockout mice were originally produced by inserting a stop codon in Exon 3 of the gene (E3stop) through embryonic stem cell-based homologous recombination. The main problems of utilizing E3stop host embryos for lung SCOG are that these animals all have a tracheoesophageal fistula (TEF), which cannot be corrected by donor stem cells, and most of them have monolateral sac-like lungs. To improve the mouse model towards achieving SCOG-based lung generation, in this project, we used the CRISPR/Cas9 tool to remove Exon 2 of the gene by zygote microinjection and successfully produced *TTF-1* knockout (E2del) mice. Similar to E3stop, E2del mice are birth-lethal due to retarded lung development with sac-like lungs and only a rudimentary bronchial tree, increased basal cells but without alveolar type II cells and blood vessels, and abnormal thyroid development. Unlike E3stop, 57% of the E2del embryos presented type I tracheal agenesis (TA, a kind of human congenital malformation) with a shortened trachea and clear separations of the trachea and esophagus, while the remaining 43% had TEF. Furthermore, all the E2del mice had bilateral sac-like lungs. Both TA and bilateral sac-like lungs are preferred in SCOG. Our work presents a new strategy for producing SCOG host embryos that may be useful for lung regeneration.

## 1. Introduction

For many end-stage diseases, organ transplantation is a last resort; however, there is a worldwide shortage of donor organs. Stem cell complementation-based organ regeneration (SCOG), albeit still at an infant stage, represents a new approach to addressing this problem [1,2]. In SCOG, the donor stem cells are injected into a host embryo that has a gene knockout that causes organ development failure by the host cells; as a result, the organ will be only/largely derived from the donor stem cells. As such, patient-specific organs may be generated, offering a potential solution to the organ shortage problem. The *Pdx1*^−/−^ pancreatogenesis-disabled model is a well-known representative of SCOG [3]. In 2010, Kobayashi et al. first used *Pdx1*^−/−^ mouse blastocysts to study interspecific stem cell complementarity and confirmed that the pancreatic vacancy was suitable for generating a functional rat pluripotent stem cell (PSC)-derived pancreas in a mouse environment [3]. Later, in 2017, Yamaguchi et al. performed the reverse experiment via creating *Pdx1*^−/−^ rat blastocysts followed by a mouse PSC complementation [4]. The pancreatogenesis-disabled model and blastocyst complementation strategy have also been extended to pigs [2,5]. In the past decade, more organ vacancy models for the heart [6,7], liver [5,8], kidneys [5,9,10], and lungs [11,12,13] have been produced with the aim of SCOG-based tissue and organ regeneration.

The primary lung vacancy mouse models were generated via full-body *Fgf10* deletion and epithelial-specific *Fgfr2* or *β-catenin* deletion [11,12]. However, the full-body *Fgf10* deletion mouse model manifested serious developmental abnormalities involving not only the lungs but also many other organs [14]. An additional disadvantage in the *Fgf10*, *Fgfr2,* or *β-catenin* deletion models is they still have parenchyma tissue, which limits donor stem cells’ ability to integrate into the host lungs [11,12]. Hence, to allow more donor stem cells to integrate into the targeted organs, a precise lung vacancy model without virtual inner tissue should be explored.

Thyroid transcription factor 1 (*TTF-1*) knockout mice have recently emerged as another potential SCOG host for lung regeneration [13,15]. TTF-1, also known as NKX2.1, is a tissue-specific transcription factor that regulates the embryonic development and differentiation of the thyroid and, more importantly, lungs [16]. *TTF-1* knockout mice were originally produced via the conventional homologous recombination approach by inserting a neomycin-resistant (*neo*^+^) gene with a stop codon in Exon 3 of the gene (E3stop) through embryonic stem cell-based genetic engineering, germline chimerism, and transmission [16]. The E3stop mice were birth lethal due to their retarded lung development and tracheoesophageal fistulae (TEF), and had thyroid absence and diencephalon deformity. In the context of lung development, sac-like lungs of translucent cystic structures with a rudimentary bronchial tree but without parenchyma tissue were observed in E3stop mice [16,17]. However, it has been reported that such sac-like lung structures were only observed in one side of the pleural cavity in most cases [16]. In addition, all E3stop mice developed congenital malformation TEF, in which a single tube failed to separate into a trachea and an esophagus [16,17]. When E3stop embryos were used as hosts, the derived animals exhibited the simultaneous generation of thyroids and lungs with parenchyma tissue after SCOG, meaning that the sac-like lungs can be rescued by donor stem cells; unfortunately, all the chimera pups died at birth [13]. It was noted that all the derived animals still had TEF, indicating that this defect cannot be corrected by the donor stem cells [13].

In this study, we used the CRISPR/Cas9 tool to remove Exon 2 of the *TTF-1* gene by zygote microinjection and successfully produced *TTF-1* knockout (E2del) mice. The E2del mice showed a series of novel phenotypes including bilateral sac-like lungs and type I tracheal agenesis (TA) with shortened trachea and the complete separation of the trachea and esophagus. The E2del mice might provide a novel development niche for lung regeneration studies and serve as a useful tool for exploring the functions of the *TTF-1* gene in embryo development.

## 2. Materials and Methods

### 2.1. Animals

All mice were housed in barrier facility with a 12 h on/12 h off light cycle. All animal experiments were approved by the Animal Care and Use Committee of the Institute of Zoology, Chinese Academy of Sciences (CAS).

### 2.2. Preparation of Cas9 mRNA and sgRNA

To target the Exon 2 region of the mouse *TTF-1* gene (GenBank, NC_000078.7), four single-guide RNAs (sgRNAs) were designed using the online CRISPR Design Tool (http://crispr.mit.edu/, accessed on 18 October 2019) and orderly named TTF1-sgRNA1 (5′-TGACATCTTGAGTCCCCTGG-3′), TTF1-sgRNA2 (5′-TTCTTGTAGCTTTCCTCCAG-3′), TTF1-sgRNA3 (5′-CGCCTACCACATGACGGCGG-3′), and TTF1-sgRNA4 (5′-CTCTCGCACTCCGCCGTGG-3′). The annealed DNA oligos of sgRNAs were cloned into the BsaI (NEB; Ipswich, MA, USA) sites of the sgRNA cloning vector (pUC57kan-T7-gRNA, Addgene, #115520; Cambridge, MA, USA) to form sgRNA-expressing plasmids (pUC57kan-T7-TTF1-sgRNAs) separately. The four sgRNA-expressing plasmids were completely linearized by restrictive digestion with DraI (NEB) and were transcribed in vitro with MEGA shortscript™ T7 Transcription Kit (Invitrogen, Ambion, AM1354; Grand Island, NY, USA), and the produced sgRNAs were purified with miRNeasy Mini Kit (Qiagen; Venlo, The Netherlands) according to the manufacturer’s instructions.

The Cas9-expressing plasmid (pST1374-N-NLS-flag-linker-Cas9, Addgene, #44758) was linearized with the restriction enzyme AgeI (NEB) and was transcribed in vitro using an mMESSAGE mMACHINE™ T7 Transcription Kit (Invitrogen, Ambion, AM1344). Then, the produced Cas9 mRNA was purified using the same method as above.

### 2.3. Microinjection

C57BL/6J female mice were superovulated with 5 IU of Pregnant Mare Serum Gonadotropin (PMSG) and 5 IU of human chorionic gonadotropin (hCG) 48 h apart [18] and mated overnight with C57BL/6J male mice. Zygotes were harvested from ampullae of superovulated females and were placed in KSOM medium. The purified Cas9 mRNA and TTF1-sgRNAs were mixed and the final concentration in the mixture was 10 ng/µL Cas9 mRNA and 5 ng/µL of each sgRNA. The mixture of Cas9 mRNA and sgRNAs were co-microinjected into the pronucleus of 1-cell zygotes. The injected zygotes were transferred into KSOM medium and cultured at 37 °C in 5% CO_2_ for 24 h and surviving 2-cell stage embryos were transferred into estrus-synchronized foster CD1 recipients via oviduct transfer. The E19 mouse embryos were obtained by surgery on the pregnant mice and dissected or fixed for further genotypic confirmation and phenotypic characterization.

### 2.4. Genotyping and Sequencing

To confirm the *TTF-1* genotype of obtained embryos, their tails were isolated and genomic DNA was extracted via TIANamp Genomic DNA Kit (TIANGEN; Peking, China) according to the manufacturer’s instructions. Via polymerase chain reaction (PCR) covering the target sites, the DNA fragments of *TTF-1* Exon 2 were amplified using Green Taq Mix (Vazyme; Nanjing, China). The forward PCR primer was 5′-GGTGTTTACCTTGTCATCAGCATGTAAGCTAATTATCTCGG-3′ and the reverse one was 5′-TCACTTACTGGCGGGGAAGCGC-3′. The PCR conditions were 94 °C for 5 min; 94 °C for 30 s, 65 °C for 30 s, and 72 °C for 2 min for 35 cycles; and then 72 °C for 7 min with the temperature held at 4 °C. To verify the presence of biallelic *TTF-1* Exon 2 deletions, the purified PCR products were cloned into pMD18-T vector (TAKARA; Kusatsu, Shiga, Japan) and transformed into DH5α-competent *E. coli* (TIANGEN). More than 10 *E. coli* colonies with positive plasmids were randomly selected for Sanger sequencing, for which the results were contrasted with wild-type.

### 2.5. Quantitative RT-PCR (qRT-PCR) Analyses

The lung tissues were lysed with RNAiso Plus (Trizol, TAKARA) and total RNA was isolated according to the manufacturer’s instructions. The cDNA was synthesized from total RNA using HiScript Q RT SuperMix for qPCR (Vazyme), and the qRT-PCR reactions were facilitated using ChamQ Universal SYBR qPCR Master Mix (Vazyme) in an ABI-7900 Real time PCR system (Applied Biosystems; Hercules, CA, USA). The qPCR conditions were 95 °C for 30 s along with 95 °C for 10 s and 60 °C for 30 s at 40 cycles; the additional dissociation stage was 95 °C for 15 s, 60 °C for 60 s, and 95 °C for 15 s. The gene-specific primers used in this study are listed in Table 1. The mRNA expression levels were calculated by the delta CT method and normalized to the relative quantity of glyceraldehyde-3-phosphate dehydrogenase (*Gapdh*) control transcripts.

### 2.6. Hematoxylin and Eosin (H&E) Staining and Immunohistochemistry (IHC) Analysis

Mouse embryos or tissues were fixed with 10% formalin and then dehydrated and embedded in paraffin. Tissue sections of 4 μm were dewaxed in xylene and rehydrated in ethanol. The nuclei were stained with hematoxylin for 3 min followed by 0.6% ammonia water treatment until the nuclei turned blue. Then, the cytoplasm was stained red with eosin for 2 min. After re-dehydration with ethanol and placement in xylene for 20 min, the tissue was sealed with resin and observed by Eclipse 80i Microscope (Nikon; Tokyo, Japan).

In IHC analysis, 30% hydrogen peroxide was used at 37 °C for 15 min to remove endogenous catalase after dewaxing tissue sections and gradient hydration. Citric acid was used at 98 °C for 20 min to repair antigens. The antigen-repaired sections were cooled to room temperature in ice water and incubated in 1% bovine serum albumin (BSA) at room temperature for 1 h for antigen blocking. After their sealing, the sections were incubated in primary antibody overnight at 4 °C, and then incubated in secondary antibody at room temperature for 2 h. DAB staining solution (Invitrogen) was used to color tissue proteins after PBST (0.05% Tween 20 in PBS, pH 7.4) cleaning. Finally, the tissue sections were redyed with hematoxylin. The primary antibodies included TTF-1 (1:300, Santa Cruz, SC53136; Dallas, TX, USA), Trp63 (1:5000, Abcam, ab124762; Cambridge, MA, USA), CD31 (1:200, Santa Cruz, SC376764), and α-SMA (1:2000, Millipore, CBL171; Darmstadt, Germany), and secondary anti-bodies included goat anti-rabbit IgG (1:10,000, Abcam, ab6721) and goat anti-mouse IgG (1:5000, KeyGene, KGAA37; Rockville, MD, USA). Images were obtained on Eclipse 80i Microscope and analysis was performed by Image J software (Fiji distribution).

### 2.7. Immunofluorescence (IF) Analysis

After dewaxing and rehydration, the tissue sections were repaired with antigens, permeated with 0.2%Triton X-100 in PBS for 20 min, blocked with 10%BSA at room temperature for 1 h, and treated with primary antibody solution at 4 °C overnight. After washing with PBS, the tissue sections were incubated in the secondary antibody solution for 1 h at room temperature. The primary antibodies included TTF-1 (1:250, Cell Signaling Technology, #12373; Beverly, MA, USA), SP-C (1:200, Santa Cruz, SC518029), Trp63 (1:200, Abcam, ab124762), CC10 (1:200, Santa Cruz, SC365992), and α-Tub (1:1000, Abcam, ab7291), and secondary antibodies included Cy3 goat anti-rabbit IgG (1:500, ABclonal, AS007; Wuhan, China), goat anti-rabbit IgG Alexa Fluor 488 (1:1000, Invitrogen, A11034), goat anti-mouse IgG Alexa Fluor 546 (1:500, Invitrogen, A11030), and goat anti-mouse IgG Alexa Fluor 488 (1:1000, Invitrogen, A32723). The nuclei were stained with DAPI (Sigma, D9542; St. Louis, MO, USA). Images were obtained on Eclipse 80i Microscope and analysis was performed by Image J software.

### 2.8. Statistical Analysis

All data are depicted as mean ± standard error of the mean (SEM). The statistical analyses were computed via GraphPad Prism 9 Software (San Diego, CA, USA). For statistical comparison between two groups, the paired Student’s *t* test was used. *p* values of less than 0.05, 0.01, 0.001, or 0.0001 were considered statistically significant and indicated as *, **, ***, or ****, respectively, in the figures.

## 3. Results

### 3.1. CRISPR/Cas9-Mediated Mouse TTF-1 Exon 2 Deletion

**Figure 1:**
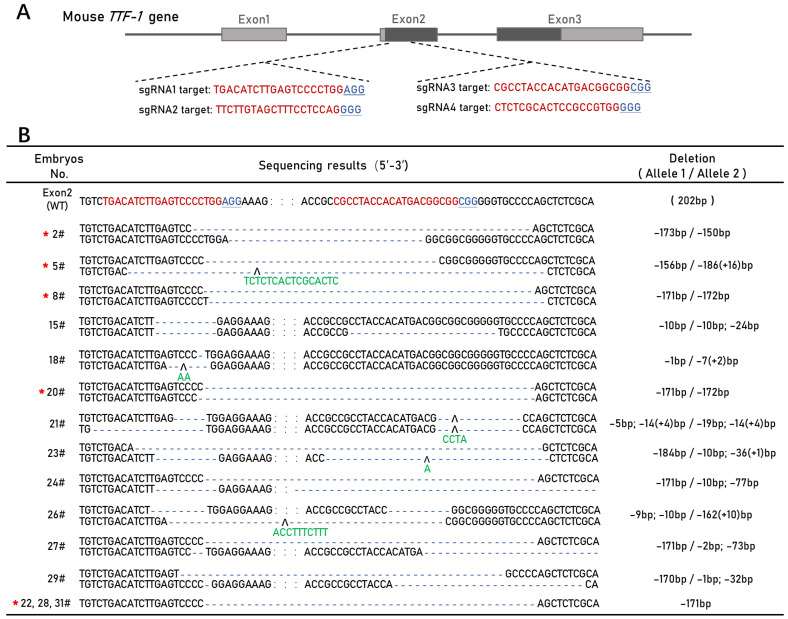
Generation of the *TTF-1* Exon 2 deletion mouse model (E2del). (**A**) Schematic diagram of mouse *TTF-1* gene and the location of designed single-guide RNAs (sgRNAs). Four sgRNAs were designed to target mouse *TTF-1* gene and located in Exon 2 (TTF1-sgRNA1 and TTF1-sgRNA2 in the forepart, TTF1-sgRNA3 and TTF1-sgRNA4 in the rear part). Coding domain sequence (CDS) region (Black), target sequence (Red), and protospacer adjacent motif (PAM) region (Underlined blue). (**B**) Genotypes of the E2del embryos with biallelic deletions. Exon 2 (WT) indicates the 202 bp *TTF-1* Exon 2 sequencing result containing the targeting sites of TTF1-sgRNA1 and TTF1-sgRNA3 in wild-type embryos. Target sequence (red), PAM region (underlined blue), omitted sequence (: : :), deleted sequence (- - -), inserted sequence (green below **∧**), and the E2del embryos with biallelic large-fragment deletions (red star).

To delete the coding region of the *TTF-1* gene in mice using CRISPR/Cas9, we designed four sgRNAs (sgRNA1, sgRNA2, sgRNA3, and sgRNA4) to target the DNA sequences in Exon 2 (Figure 1A). We cloned each of the four sgRNAs into an sgRNA cloning vector and constructed four sgRNA-expressing plasmids separately. The linearized sgRNA-expressing plasmids and Cas9-expressing plasmid were transcribed and purified in vitro.

First, to determine the editing efficiency of each sgRNA, the Cas9 mRNA and four TTF1-sgRNAs were co-microinjected into the mouse zygotes. Based on the PCR amplification and Sanger sequencing of the region containing the target sites, both TTF1-sgRNA1 and TTF1-sgRNA3 had higher gene-editing activity at approximately 88.89% and 100% in the biallelic modified embryos (the sequencing results and comparison are shown in Supplemental Tables S1 and S2).

Next, to produce *TTF-1* gene knockout mice with Exon 2 deletion, we mixed and microinjected the in vitro-transcribed Cas9 mRNA, TTF1-sgRNA1, and TTF1-sgRNA3 into zygotes, followed by embryo transfer to estrus-synchronized foster mothers. At E19, from eight gravid mice, we collected 32 mouse embryos and detected their *TTF-1* gene sequences. Most (27 of 32, 84.38%) embryos were found to have the biallelic *TTF-1* Exon 2 edited (E2del embryos). Among these 27 E2del embryos, 7 had biallelic large-fragment deletions (7/32, 21.88%). A total of 15 E2del embryos were used for phenotype analysis in the following experiments, and their genotypes are listed in Figure 1B. All the E2del embryos are similar to their wild-type siblings but with various degrees of bloodshot in their overall external physical characteristics (Supplemental Figure S1A), and each successively died within 20 min.

### 3.2. The E2del Mouse Embryos Displayed Bilateral Sac-like Lungs

**Figure 2:**
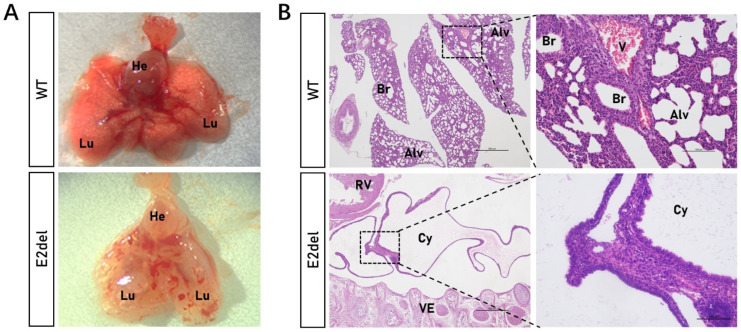
Gross morphology of the *TTF-1* Exon 2 deletion (E2del) lungs. (**A**) Pulmonary morphology of the E2del mice at E19 compared to the age-matched wild-type control. (**B**) H&E staining results of lung sections in the wild-type and the E2del embryos. Scale bar: primitive is 500 μm, and enlarged is 100 μm. WT, wild-type control; E2del, *TTF-1* Exon 2 deletion mice; He, heart; Lu, lung; Br, bronchus; Alv, alveoli; V, blood vessel; Cy, cyst; RV, right ventricle.

**Figure 3:**
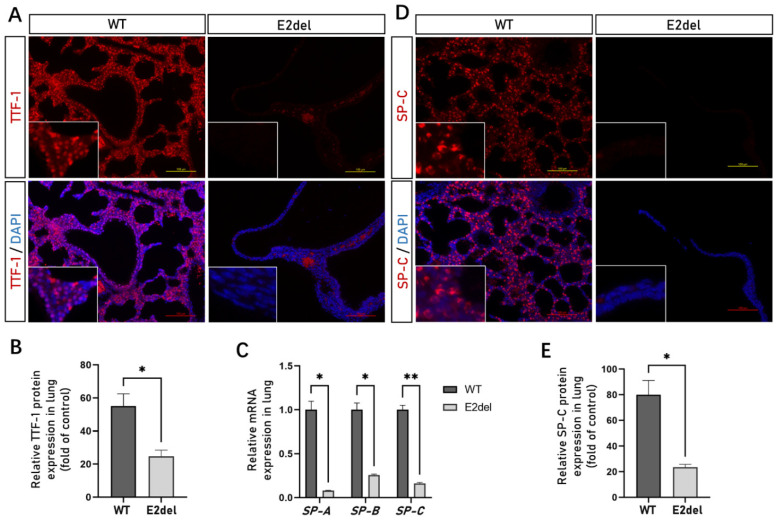
The specific protein and gene expression of alveolar cells in the E2del lungs. (**A**) The expressions of TTF-1 protein in the wild-type and the E2del lungs at E19 as determined by IF staining. Scale bar: 100 μm. (**B**) The means of data of TTF-1-positive area as in A. (**C**) The relative mRNA expression of *SP-A*, *SP-B*, and *SP-C* genes showed that specific genes of alveolar type II cells were significantly inhibited in the E2del lungs. (**D**) The expressions of SP-C protein in the wild-type and the E2del lungs at E19. Scale bar: 100 μm. (**E**) The means of data of SP-C-positive area as in (**D**). The data are presented as mean ± SEM. * *p* < 0.05 and ** *p* < 0.01 via paired Student’s *t* test.

The *TTF-1* gene is the first homeodomain transcriptional factor expressed in primitive respiratory epithelial progenitors with a critical role in the morphogenesis of the lungs [16]. First, we dissected the pleural cavities of more than 10 dead E2del embryos and found that all of them had translucent cystic lungs (Figure 2A). To explore the organizational structure of the cystic lungs, we fixed the entire set of whole E2del embryos (No. 8, 20, 21, 22, 23, 24, 26, 27, 28, and 29#). The H&E staining showed that all the E2del mice had dilated sac-like structures in their pleural cavities and displayed this phenotype in both pleural cavities (Figure 2B and Supplemental Figure S1B). Compared with their wild-type siblings, the abnormal sac-like structures of the E2del mice lacked bronchi, lung alveoli, and blood vessels, which were replaced by a rudimentary bronchial tree including abnormal bronchial epithelia with fuzzy boundaries. Then, the IF analysis of the whole-fixed mouse embryos showed that the dilated sac-like lungs did not contain any alveolar cells with TTF-1 protein (Figure 3A,B).

### 3.3. The E2del Lungs Lacked Alveolar Type II Cells

TTF-1 is essential for the expression of the *SP-A* (surfactant protein A), *SP-B,* and *SP-C* genes determining alveolar type II cells in the lungs [19]. To investigate the molecular defects caused by *TTF-1* Exon 2 deletion during lung development, we examined the mRNA expression of *SP-A*, *SP-B,* and *SP-C* genes in the E2del lungs of No. 2, 15, and 18# embryos. Compared with the normal embryos, the relative expressions of *SP-A*, *SP-B,* and *SP-C* genes were significantly inhibited (Figure 3C). As observed through an IF analysis, the SP-C protein almost disappeared in the E2del lungs while it was stably expressed in the normal lungs (Figure 3D,E). *PDPN* (podoplanin, also known as T1α) mRNA was detected to be expressing in only alveolar type I cells and was also downregulated in the E2del lungs. The regulated genes *LAMP3* (lysosomal-associated membrane protein 3) and *ABCA3* (ATP binding cassette transporter A3) were inhibited by the *TTF-1* gene’s inactivation (Supplemental Figure S2A). These results suggest that the lungs with biallelic *TTF-1* Exon 2 deletion lacked alveolar type II cells and contained reduced alveolar type I cells during embryonic development.

### 3.4. The E2del Lungs Contained Abnormally Abundant Basal Cells

**Figure 4:**
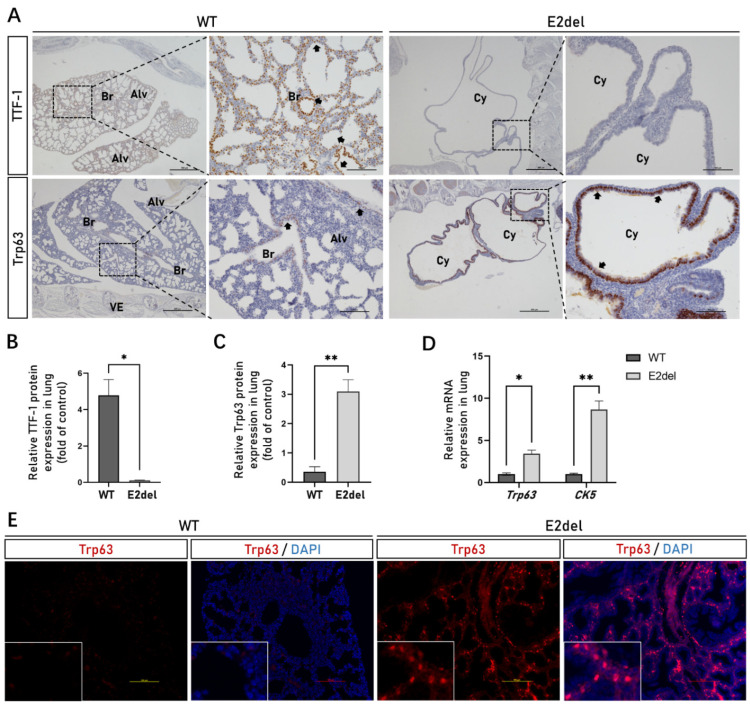
The specific protein and gene expression of basal cells in the E2del lungs. (**A**) The expressions of TTF-1 protein in the wild-type and the E2del lungs at E19 as determined by IHC staining. Scale bar: primitive is 500 μm, enlarged is 100 μm. Br, bronchus; Alv, alveoli; Cy, cyst; VE, vertebra. The arrowheads indicate protein-positive area. (**B**) The means of data of TTF-1-positive area as in A. (**C**) The means of data of Trp63-positive area as in A. (**D**) The relative mRNA expression showed that specific genes of *Trp63* and *CK5* genes of basal cells were significantly upregulated in the E2del lungs. (**E**) The expressions of Trp63 protein in the wild-type and the E2del lungs at E19 as determined by IF staining. Scale bar: 100 μm. The data are presented as mean ± SEM. * *p* < 0.05, ** *p* < 0.01, paired student’s *t* test.

Both *Trp63* (transformation related protein 63) and *CK5* (keratin 5) genes should be characteristically expressed in basal cells, which partly make up the pseudostratified mucociliary epithelium of the lungs and are stem cells of the tracheal epithelium [20]. In our research, the IHC and IF analyses of the whole-fixed mouse embryos confirmed that the sac-like lung tissues of the E2del embryos contained few alveolar cells expressing the TTF-1 protein (Figure 4A,B), while their inside structures consisted of basal cells highly expressing the Trp63 protein (Figure 4A,C,E and Supplemental Figure S2B). The results of the quantitative RT-PCR showed that the expression levels of the *Trp63* and *CK5* genes were both considerably upregulated in the E2del lung tissues (No. 2, 15 and 18#; Figure 4D). Presumably, as stem cells, the basal cells failed to develop into tracheal epithelium, and, consequently, formed a sac-like lung structure.

### 3.5. The Abnormal Development of the Other Functional Cells in the E2del Lungs

**Figure 5:**
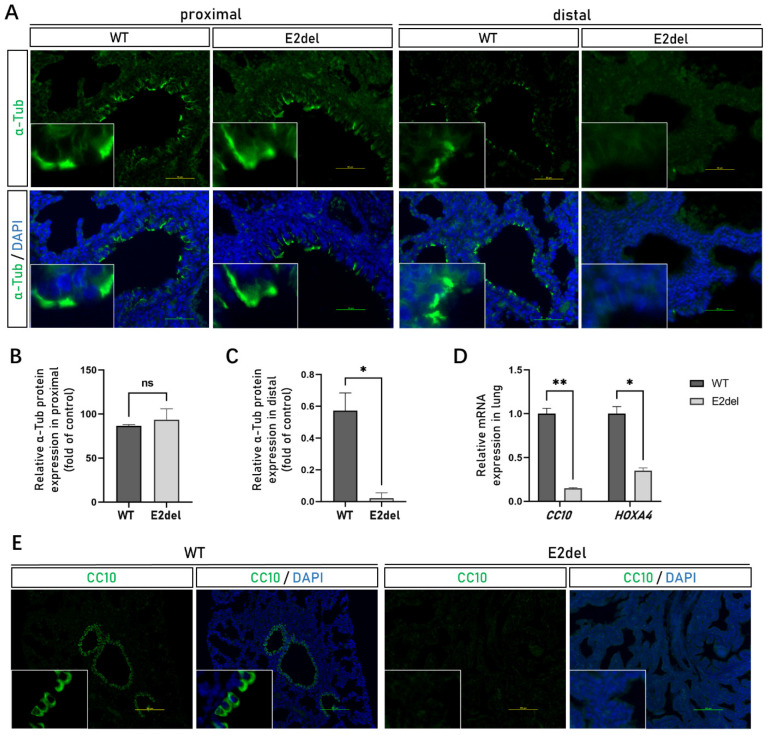
The specific proteins and genes expression of ciliated cells, Clara cells and mesenchymal cells in the E2del lungs. (**A**) The expressions of α-Tub protein in the proximal and distal regions of the wild-type and the E2del lung bronchus at E19 as determined by IF staining. Scale bar: 50 μm. (**B**) The means of data of α-Tub-positive area as in proximal bronchus of A. (**C**) The means of data of α-Tub-positive area as in distal bronchus of A. (**D**) The relative mRNA expression of *CC10* and *HOXA4* genes showed that specific genes of Clara cells and mesenchymal cells were expressed at a low level in the E2del lungs compared with the wild-type lungs. (**E**) The expression of CC10 protein in the wild-type and the E2del lung parenchyma at E19 as determined by IF staining. Scale bar: 100 μm. The data are presented as mean ± SEM. ns denotes no significance. * *p* < 0.05 and ** *p* < 0.01 via paired Student’s *t* test.

The ciliated cells are functional epithelial cells in the proximal and distal regions of lung bronchi and express the cytoskeletal protein α-Tubulin (α-Tub) [21,22]. Compared with the normal lungs analyzed by IF staining, the E2del lungs had no significant difference with respect to the expression of the α-Tub protein in the ciliated cells of the proximal main bronchus and lobar bronchus (Figure 5A,B). Conversely, the expression of the α-Tub protein was significantly down-regulated in the ciliated cells of the distal bronchus and the terminal bronchus of the E2del lungs (Figure 5A,C). These results suggest that the branching morphogenesis of the epithelium was blocked, and that the E2del embryos failed to form bronchial trees during E10.5 and E16.5.

Furthermore, the Clara cell 10-kDa protein (CC10) is thought to be secreted by a certain kind of Clara cells and essential for maintaining airway patency in the lung parenchyma [23]. We found that the expression of the *CC10* gene was inhibited (Figure 5D) and the cells containing CC10 protein mostly disappeared in the E2del lungs (Figure 5E and Supplemental Figure S2C). *HOXA4* (homeobox A4) gene representing embryo mesenchyme was expressed at a low level in the E2del lungs compared with wild-type lungs (Figure 5D). The IHC analysis showed that endothelial cells containing CD31 (endothelial cell adhesion molecule-1) and smooth muscle cells containing α-SMA (alpha-smooth muscle actin) had few differences among the E2del and wild-type lungs (Supplemental Figure S3D).

### 3.6. The E2del Mouse Embryos Showed Malformation Type I TA or TA/TEF

**Figure 6:**
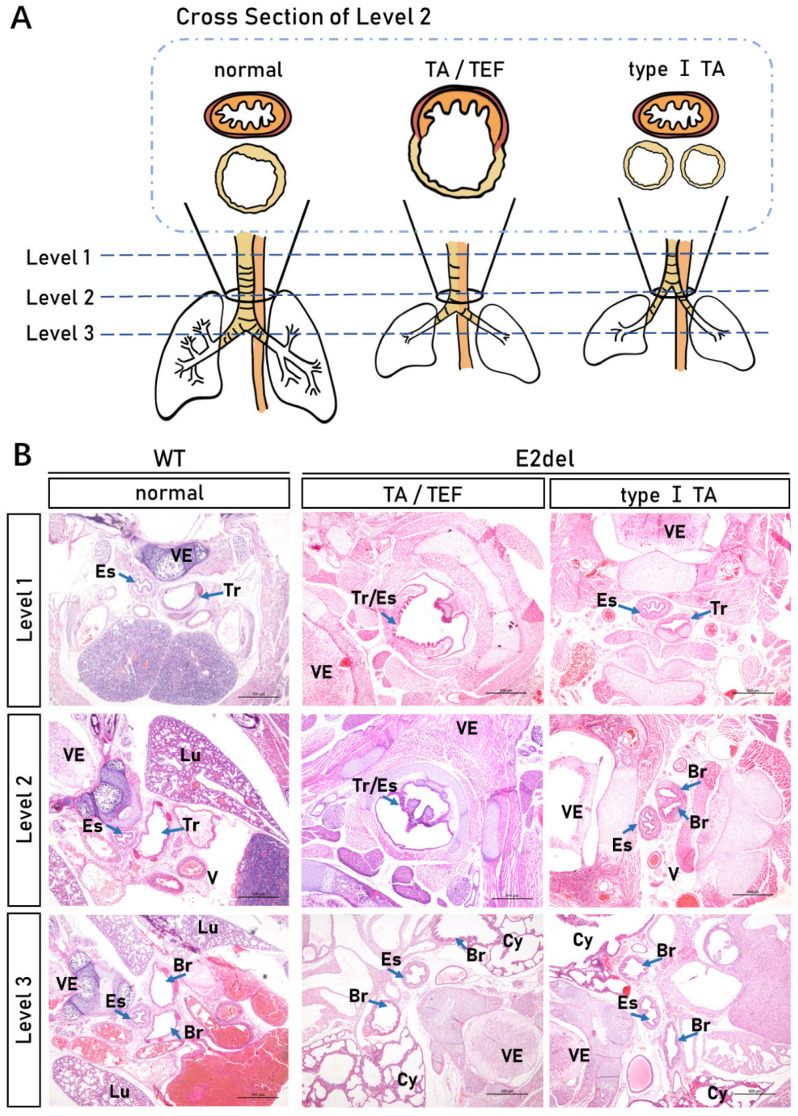
Tracheal and esophageal malformation in the E2del mouse embryos. (**A**) Schematic diagram of tracheal and esophageal malformation of the E2del embryos. From head and neck, mice were divided into three levels (Level 1, Level 2, and Level 3) of transverse views to detect septation between the trachea and the esophagus. The normal, TA/TEF, and type I TA indicate the morphology of the wild-type and two types-E2del embryos in the cross section of Level 2. (**B**) H&E staining results of tracheal and esophageal sections in the wild-type and E2del embryos. Scale bar: 500 μm. VE, vertebra; Tr, trachea; Es, esophagus; Lu, Lung; V, blood vessel; Br, bronchus; Cy, cyst. The arrowheads indicate specific tissues.

The *TTF-1* mutation in the E3stop mice resulted in a congenital TEF malformation [16,17]. In our study, seven randomly selected E2del embryos were examined for tracheal and esophageal malformation. To detect septation between the trachea and the esophagus, we compared the transverse views of the E2del and the wild-type mice at three levels (Level 1, Level 2, and Level 3) from head to neck. The results of the H&E staining showed that No. 20, 23, 24, and 28# E2del mice had shortened tracheas with their bottoms moved upwards from Level 3 to Level 2, similar to human congenital malformation type I TA (Figure 6A,B). Whereas the No. 21, 27, and 29# E2del mice had similar shortened tracheas as in TA, while their tracheoesophagi were not separated into a trachea and esophagus in the regions of both Level 1 and Level 2, termed TA/TEF (Figure 6A,B).

### 3.7. The E2del Mouse Embryos Had Thyroid Developed and Diencephalon Abnormalities

**Figure 7:**
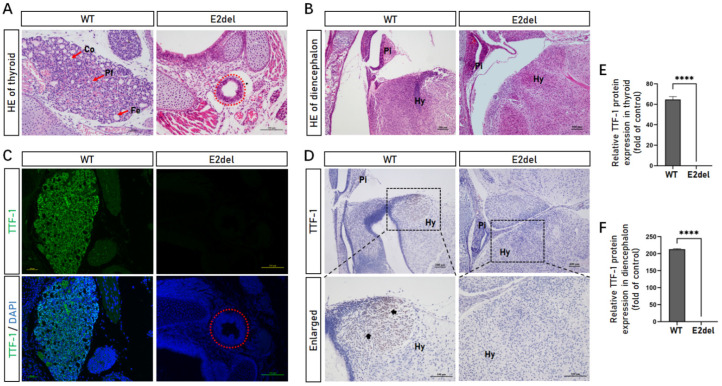
The morphology and TTF-1 protein expression of thyroid and diencephalon in the E2del mouse embryos. (**A**) H&E staining results of thyroids in the wild-type and the E2del embryos at E19. Scale bar: 50 μm (WT) and 100 μm (E2del). Co, colloid; Pf, parafollicular cells; Fe, follicular epithelial cells. The arrowheads indicate specific cells. The red dashed circle denotes the thyroid lacking inner tissue such as follicles and peripheral cells. (**B**) H&E staining results of diencephalons in the wild-type and the E2del embryos at E19. Scale bar: 100 μm. Pi, pituitary; Hy, hypothalamus. (**C**) The expressions of TTF-1 protein in the wild-type and the E2del thyroid at E19 as determined by IF staining. Scale bars: 50 μm (WT) and 100 μm (E2del). The red dashed circle points out the thyroid in the E2del embryos. (**D**) The expressions of TTF-1 protein in the wild-type and the E2del diencephalon at E19 as determined by IHC staining. Scale bars: 100 μm. Pi, pituitary gland; Hy, hypothalamus. The arrowheads indicate TTF-1-positive cells (stained brown). (**E**) The means of data of TTF-1-positive area as in C. (**F**) The means of data of TTF-1-positive area as in D. The data are presented as mean ± SEM. **** *p* < 0.0001, as determined by paired Student’s *t* test.

In addition to the respiratory system, TTF-1 is expressed in the thyroid and diencephalon and participates in their differentiation, development, and functional maintenance. To assess the effects of the *TTF-1* Exon 2 deletion on the thyroid and diencephalon, we sectioned the necks and brains of the E2del mouse embryos. The H&E staining showed that the E2del embryos (No. 20, 22, 24, and 29#) still had thyroids developed but lacked inner tissue, such as follicles and peripheral cells, relative to the normal mouse embryos (Figure 7A). Concurrently, compared with the normal embryos, the diencephalons of the E2del embryos (No. 5, 23, 24, 26, and 27#) had no significant differences in their tissue organization (Figure 7B). The IF and IHC analysis confirmed that the thyroids and diencephalons of the E2del mice did not express TTF-1 protein (Figure 7C–F).

## 4. Discussion

TTF-1 was originally identified as a transcription factor. The protein structure and function of TTF-1 are extremely similar in humans, rats, and mice [19]. Four transcripts were found during the fetal development and cell fate decision in mice. The *TTF-1* gene’s expression is incredibly complicated, typically displays spatiotemporal inputs from transcripts that are less distinctive, and sequentially confines progenitors to certain tissue lineages for an organism’s development [24,25]. During early embryogenesis, TTF-1 is essential for the organogenesis of the lungs and thyroid [16].

In our study, we chose to delete exon 2 of *TTF-1* (E2del) to generate *TTF-1* knockout mice, which is a different process from the insertion of the ~1600 bp *neo* sequence in exon 3 of *TTF-1* (E3stop) in the previous *TTF-1*^−/−^ murine model [16] (Supplemental Figure S3). Consistent with the E3stop embryos, the E2del embryos displayed sac-like lungs. Differing from the E3stop embryos with the sac-like lungs in only the right or left cavity, the sac-like lungs appeared in both pleural cavities in the E2del embryos. Due to the bilateral lung development block, the lung development of E2del embryos should be arrested at the inception of the pseudo glandular period. Many TTF-1-responsive genes during lung development have been defined. TTF-1 determines alveolar type II cells by affecting the expression of *SPs* (surfactant-A/B/C), *CC10*, *ABCA3,* and *LAMP3* genes [19,26]. Our results showed that these genes were commonly inhibited in the E2del lungs. The expression level of α-Tub protein in the distal bronchus and terminal bronchus was decreased, which resulted in the failure of the formation of intact bronchial trees in the E2del pups. Besides the expression characteristics of the above genes, the *Trp63* proteins of basal cells were abnormally expressed and lined most of the epithelial surfaces of the cystic structures in E2del similar to those in the E3stop lungs [15]. The specific gene of embryo mesenchyme *HOXA4* was lowly expressed in the E2del lungs; this is different from its normal expression throughout lung development in the E3stop embryos [17]. The TTF-1 Exon 2 deletion-derived gene network disorder led to the abnormally developed lungs lacking alveolar type II cells and Clara cells, while only a rudimentary bronchial tree and fewer functional cells (slight amounts of alveolar type I cells, bronchial epithelial cells, mesenchymal cells and certain amounts of endothelial cells and smooth muscle cells) remained. The stem cell complementation with E3stop blastocyst as a host proved that the alveolar cells were almost entirely descendent from donor ESCs, albeit the endothelial, immune, and stromal components were mosaic [13]. Based on the above results and analysis, we expect that E2del will provide pulmonic vacant niches with which to generate lungs mainly derived from donor stem cells.

During mid-gestation, the tracheal and the esophageal tubes arise from a common endodermal origin: the anterior foregut tube. The disruption of the complex set of morphogenetic events can result in severe birth defects, such as TEF, in which the lumina of the trachea and esophagus remain connected [27]. The E3stop embryos had severe the birth defects and complete TEF since the normal epithelial-mesenchymal interactions were disrupted [17]. There are significant differences in the composition of the epithelium between the trachea and the esophagus [28,29]. In the trachea, the mesenchyme develops C-shaped cartilage rings ventrally and the trachealis muscle dorsally. In the esophagus, the stratified squamous cells and the esophageal mesenchyme develop into concentric rings of esophageal smooth muscle. TTF-1 regulates multiple respiratory-specific genes to establish the ventral foregut domain and separate the trachea and esophagus. TA is a rare congenital anomaly in which the tracheal segment between the cricoid cartilage and the carina is absent or severely stunted and classified into three types [30]. In our study, more than half of the E2del embryos showed the type I TA with a shortened trachea and partial development of the distal trachea. The type I TA abnormality is speculated to be more favorable for donor stem cell compensation than TEF. The TEF abnormality in E3stop embryos had not been rescued by mESC complementation [13].

TTF-1 is expressed in the thyroid, pituitary gland, hypothalamus, and other diencephalic tissues, and participates in normal fetal development [16,31]. In E3stop embryos, the thyroid rudiment was not found along the trachea, and extensive abnormalities were present in the ventral forebrain and pituitary gland [16]. In contrast, the thyroids were developed in the E2del mice, albeit short of inner tissue, such as follicle-structure. The diencephalons in the E2del mice did not show obvious abnormalities. The stem cell complementation research verified that the donor cells regenerated the thyroid but did not rescue the lack of neuronal progenitors in the forebrain [13]. The developed thyroid and the normally developed other organs, such as the diencephalon, indicated that our E2del embryos might be more suited as a pulmonic regeneration niche, and our further investigations are still ongoing.

Certainly, due to their major differences in body size, gestation duration, and physiological characteristics from humans, rodents are not the optimal animal host for primate organ regeneration. However, stem cell complementation in a mouse model can still provide insights for large livestock species. Therefore, our study of E2del mice suggests the possible extension of this research to large livestock species to produce a more suitable lung vacant host in the future.

## Figures and Tables

**Table 1:** The gene-specific primers used in qRT-PCR analyses.

Gene	Sequence (5′-3′)		Length (bp)
*SP-A*	Forward	TTCCACCAATGGGCAGTCAG	192
	Reverse	GAAGCCCCATCCAGGTAGTG	
*SP-B*	Forward	GGCCTCACACTCAGGACTTC	110
	Reverse	CAGGCACTTGGGGATCACG	
*SP-C*	Forward	ATACTGAGATGGTCCTTGAGATG	135
	Reverse	GCCGCTGGTAGTCATACAC	
*PDPN*	Forward	AGAGAACACGAGAGTACAACCA	99
	Reverse	CGTTTCATCCCCTGCATTATCT	
*LAMP3*	Forward	CATCACCAGCCAAGATCGGA	155
	Reverse	AGATGCATGGGTTAGGCTGG	
*ABCA3*	Forward	GGGTGATGGACCCAACGAAT	155
	Reverse	TGCCACCATCTTCCATTCCC	
*CC10*	Forward	GAGGCCCTCCTCATGGAATC	130
	Reverse	TCCTGGTCTCTTGTGGGAGG	
*HOXA4*	Forward	GCTCTCGAACCGCCTATACC	192
	Reverse	TCGCATCTTGGTGTTGGGAA	
*Trp63*	Forward	TGCGTCGGAGGAATGAAC	160
	Reverse	ATACTTGCTGCTTTCTGATGC	
*CK5*	Forward	TCCAGTGTGTCCTTCCGAAGT	223
	Reverse	TGCCTCCGCCAGAACTGTA	
*Gapdh*	Forward	GCCTTCCGTGTTCCTACC	101
	Reverse	GCCTGCTTCACCACCTTC	

## Data Availability

Not applicable.

## Supplementary Materials

The following supporting information can be downloaded at: https://www.mdpi.com/article/10.3390/cells11233874/s1. Figure S1: Gross morphology of E2del mice and their lungs. Figure S2: The specific genes and proteins expression in the E2del lungs. Figure S3: Schematic diagram of *TTF-1* gene in E3stop mice, wild-type mice, and E2del mice. Table S1: Sequencing results of the *TTF-1* gene of the mouse embryos edited by four sgRNA-expressing plasmids in vivo. Table S2: Comparison of *TTF-1* gene editing activity of four sgRNA-expressing plasmids in vivo.
