# Supplementary material for: Development of a Lung Vacancy Mouse Model through CRISPR/Cas9-Mediated Deletion of Thyroid Transcription Factor 1 Exon 2
Source: Cells. 2022 Dec 1;11(23):3874. doi: 10.3390/cells11233874 (PMC9740088; doi:10.3390/cells11233874)
Supplement: Supplementary file 1 [file cells-11-03874-s001.zip › cells-2032068-supplementary.pdf]

Table S1. Sequencing results of the *TTF-1* gene of the mouse embryos edited by four sgRNA-expressing plasmids in vivo.

Embryos No.	Sequencing results（5′-3′)
Exon 2 (WT)	TGTCTGACATCTTGAGTcccCTGGAGGaaagctacaagaaAGTGG : : : : :<br>ACCGCCGCCTACCACATGACGGCGGCGGGGGTGCCCCAGctctcgcactccgccgtgggggGCTA
Z1	TGTCTGACATCTT----------------GAGGaaagctacaagaaAGTGG : : : : : ACCGCCGCCTACCACATGA--<br>cgcactccgccgtgggggGCTA
Z3	TGTCTGACATCTTGAGT---//--<br>cactccgccgtgggggGCTA
Z4	TGTCTGACATCTTGAGTcccC---//------------------------------------<br>CGGCGGGGGTGCCCCAGctctcgcactccgccgtgggggGCTA
Z6	TGTCTGACATCTTGAGTcccC---//------------------------------------<br>CGGGGGGGGTGCCCCAGctctcgcactccgccgtgggggGCTA
Z8	TGTCTGACATCTT----------------GAGGaaagctacaagaaAGTGG : : : : : ACCGCCGCCTACCA--A---A--------------------------------A--<br>ctctcgcactccgccgtgggggGCTA
Z9	TGTCTGACATCTTGAGTcccC---//------------------------------------<br>CGGGGGGGGTGCCCCAGctctcgcactccgccgtgggggGCTA
Z12	TGTCTGACATCTTGAGTcccC---//------------------------------------<br>CGGCGGGGGTGCCCCAGctctcgcactccgccgtgggggGCTA
Z13	TGTCTGACATCTTGAG-------TGGAGGaaagctacaagaaAGTGG : : : : : ACCGCCGCCTACCACATGACGG-----------------T---------------------<br>tccgccgtgggggGCTA
Z15	TGTCTGACATCTTGA--------------AGGaaagctacaagaaAGTGG : : : : : ACCGCC--<br>GctctcgcactccgccgtgggggGCTA

In the *TTF-1* sequences, "TGACATCTTGAGTcccCTGG" is the target of sgRNA1, "CTGGAGGaaagctacaagaa" is the target of sgRNA2,

“CGCCTACCACATGACGGCGG” is the target of sgRNA3, “ctctcgcactccgccgtgg” is the target of sgRNA4.

Table S2. Comparison of *TTF-1* gene editing activity of four sgRNA-expressing plasmids in vivo.

**No. of Embryos analyzed**	**No. of Embryos with *TTF-1* gene edited (editing activity%)**			
	sgRNA1 targeted	sgRNA2 targeted	sgRNA3 targeted	sgRNA4 targeted
9	8 (88.89%)	6 (66.67%)	9 (100%)	0

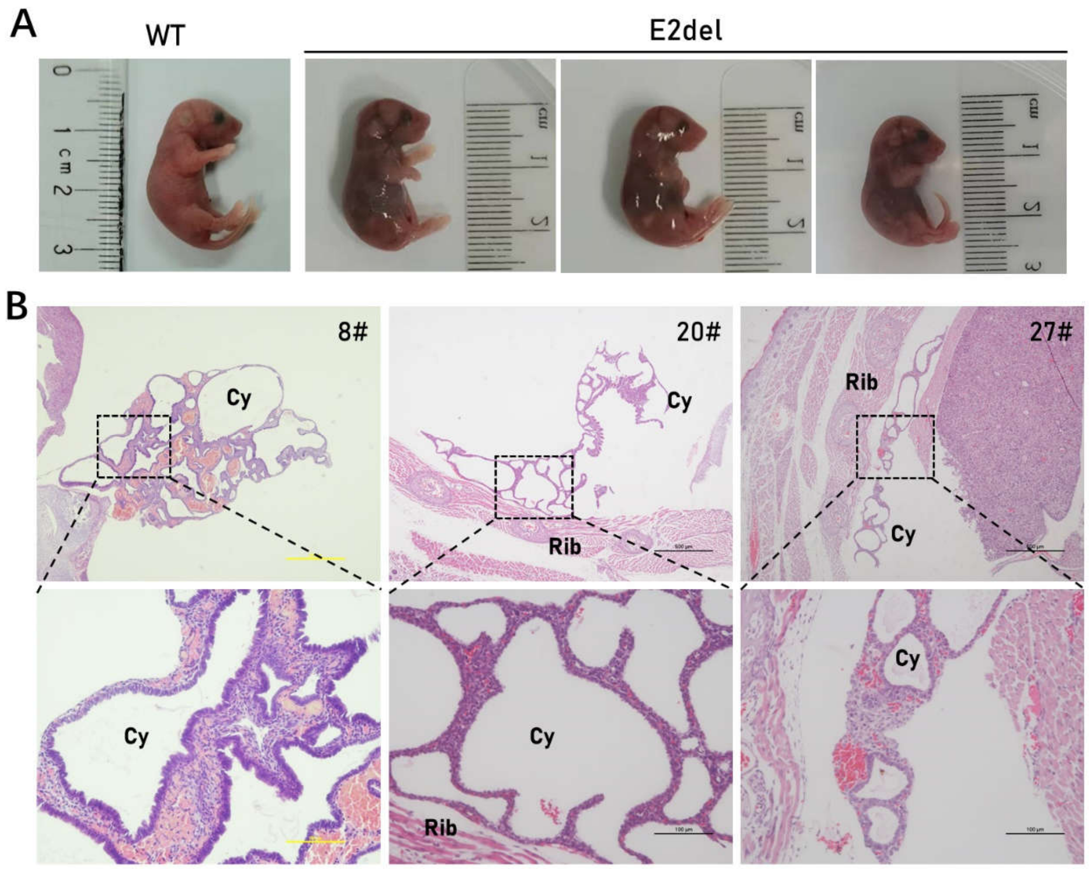

Figure S1. Gross morphology of the E2del mice and their lungs. (A) Entirety morphology of the E2del mice at E19 as compared with the age-matched wild-type control. (B) Supplemental H&E staining results of lung sections in the E2del mouse embryos. Scale bar: primitive is 500 μm, enlarged is 100 μm. Cy, cyst.

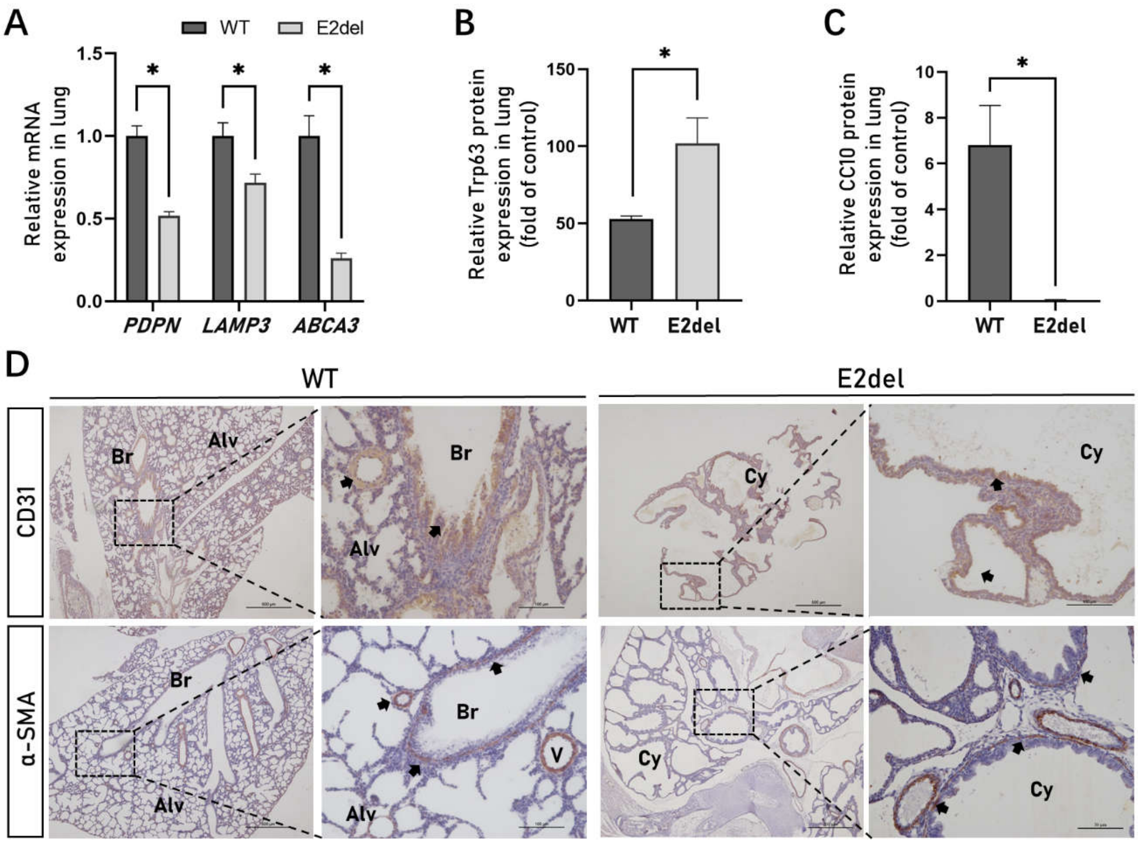

Figure S2. The specific gene and protein expression in the E2del lungs. (A) The relative mRNA expression showed that specific PDPN gene of alveolar type I cells and TTF-1-regulated gene *LAMP3* and *ABCA3* were inhibited in the E2del lungs compared to the wild-type lungs. (B) The means of data of Trp63-positive area as in figure 4E. (C) The means of data of CC10-positive area as in figure 5E. (D) The expressions of CD31 and α-SMA protein in the wild-type and E2del lungs at E19 as determined by IHC staining. Scale bar: primitive is 500 μm, enlarged is 100 μm. Br, bronchus; Alv, alveoli; Cy, cyst; V, blood vessel. The arrowheads indicate protein-positive area. The data are presented as mean ± SEM. *$p < 0.05$, via paired student's t test.

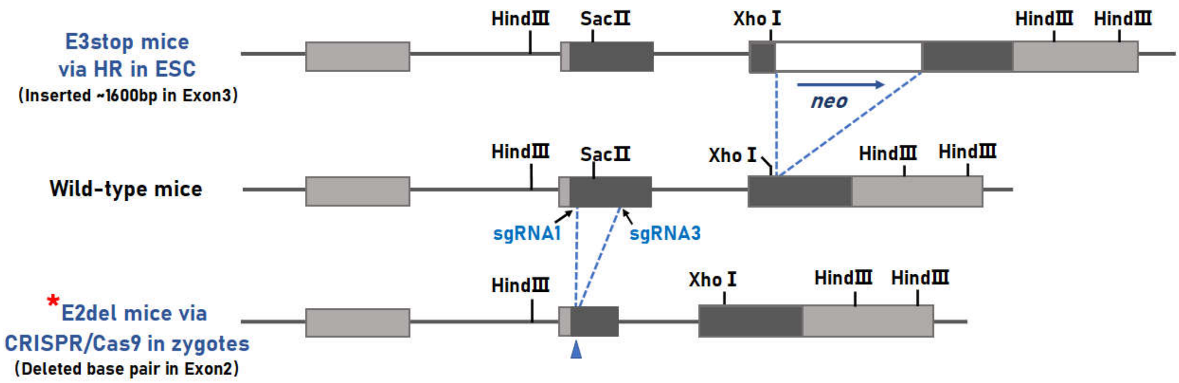

Figure S3. Schematic diagram of *TTF-1* gene in the E3stop mice, wild-type mice and E2del mice. The *TTF-1* gene of the E3stop mice was inserted the ~1600bp *neo* (neomycin) sequence in Exon 3 via HR (homologous recombination) in ESC (embryonic stem cell). The *TTF-1* gene of the E2del mice in this study (Red asterisk) was deleted base pair among the targeting sites of TTF1-sgRNA1 and TTF1-sgRNA3 in Exon 2 via CRISPR/Cas9 in zygotes.
